# Circulating TNF-like protein 1A (TL1A) is elevated early in rheumatoid arthritis and depends on TNF

**DOI:** 10.1186/s13075-020-02198-9

**Published:** 2020-05-07

**Authors:** Yun-Jeong Song, In Ah. Choi, Françoise Meylan, M. Kristen Demoruelle, Taylor Farley, Arianne C. Richard, Eric Hawley, John Botson, Yoo Jin Hong, Eun Young Lee, Sabina R. Mian, Bartlett C. Hamilton, Geoffrey M. Thiele, Ted R. Mikuls, Naveen Gara, Chris D. Ward, Sarah Lamberth, Kevin D. Deane, Theo Heller, Michael M. Ward, David M. Lee, Thi-Sau Migone, William Stohl, James R. O’Dell, Jill M. Norris, V. Michael Holers, Peter Gregersen, Yeong-Wook Song, Richard M. Siegel

**Affiliations:** 1 Immunoregulation Section, Autoimmunity Branch, NIAMS, NIH, Bethesda, MD USA; 2 Division of Rheumatology, Department of Internal Medicine, MMBS, Medical Research Center, Seoul National University College of Medicine, Seoul, Korea; 3 Division of Rheumatology, University of Colorado School of Medicine, Aurora, CO 80207 USA; 4 Division of Rheumatology, Department of Medicine, Los Angeles County University of Southern California Medical Center and University of Southern California Keck School of Medicine, Los Angeles, CA USA; 5 Rheumatology Division, Department of Medicine, University of Nebraska Medical Center and VA Nebraska-Western Iowa Health Care System, Omaha, NE USA; 6 Liver Diseases Branch, NIDDK, NIH, Bethesda, MD USA; 7 Human Genome Sciences, Rockville, MD USA; 8 Immunology Biomarkers group, Pharmaceutical Companies of J&J, LLC, Spring House, PA USA; 9 Clinical Trials and Outcomes Branch, NIAMS, NIH, Bethesda, MD USA; 10 Brigham and Women’s Hospital, Harvard Medical School, Boston, MA USA; 11 Present address: Janssen Research and Development, Spring House, PA USA; 12 Colorado School of Public Health, Aurora, CO USA; 13 Center for Genomics & Human Genetics, The Feinstein Institute for Medical Research, Hofstra North Shore-LIJ School of Medicine, Manhasset, NY USA

**Keywords:** Rheumatoid arthritis, Cytokines, Tumor necrosis factor-like cytokine 1A, TNFSF15, Collagen-induced arthritis

## Abstract

**Background:**

The tumor necrosis factor (TNF) superfamily cytokine TNF-like protein 1A (TL1A) and its receptor DR3 are essential for diverse animal models of autoimmune disease and may be pathogenic in rheumatoid arthritis (RA). However, the relationship of TL1A to disease duration, activity, and response to anti-TNF and other therapies in RA is not clear.

**Methods:**

We measured soluble TL1A in synovial fluid (SF), serum, or plasma from RA first-degree relatives (FDRs) and in early RA and established disease. We measured the effects of anti-TNF and methotrexate (MTX) therapy on circulating TL1A from multiple independent RA treatment trials. We also determined the ability of a blocking anti-TL1A antibody to inhibit clinical disease and articular bone destruction in the murine collagen-induced arthritis (CIA) model of human RA.

**Results:**

Soluble TL1A was specifically elevated in the blood and SF of patients with RA compared to patients with other diseases and was elevated early in disease and in at-risk anti-cyclic citrullinated peptide (CCP) (+) first-degree relatives (FDRs). Therapeutic TNF inhibition reduced serum TL1A in both responders and non-responders, whereas TL1A declined following MTX treatment only in responders. In murine CIA, TL1A blockade was clinically efficacious and reduced bone erosions.

**Conclusions:**

TL1A is specifically elevated in RA from early in the disease course and in at-risk FDRs. The decline in TL1A after TNF blockade suggests that TL1A levels may be a useful biomarker for TNF activity in RA. These results support the further investigation of the relationship between TL1A and TNF and TL1A blockade as a potential therapeutic strategy in RA.

## Introduction

Therapies that block tumor necrosis factor (TNF) have dramatically improved patient outcomes in several autoimmune diseases, particularly rheumatoid arthritis (RA). However, as many as 40% of RA patients have inadequate responses to TNF blockade and are in need of alternative therapies [1, 2]. Other members of the TNF superfamily of cytokines, especially those that costimulate B or T cell activation, may also be potential candidates for therapeutic blockade in RA [3]. Among these is TNF-like ligand 1A (TL1A, TNFSF15), a TNF-family cytokine expressed by immune and endothelial cells in response to a number of pro-inflammatory stimuli. Through its receptor death receptor 3 (DR3, TNFRSF25), which is expressed primarily on lymphocytes, TL1A stimulates T cell activation, proliferation, and production of a broad array of cytokines [4–6]. Deficiency in DR3, TL1A, or treatment with TL1A blocking antibodies ameliorates a broad array of autoimmune disease models, including experimental allergic encephalomyelitis, allergic lung hypersensitivity, experimental arthritis, and inflammatory bowel disease [4, 7–12].

TL1A is a candidate cytokine for mediating inflammatory effects downstream of innate immune stimuli in RA. Fc receptor (FcR) crosslinking by immune complexes and rheumatoid factor (RF) can induce TL1A expression on monocytes and dendritic cells [13, 14]. Unlike FcR crosslinking, TLR stimulation has been found by some but not other studies to stimulate TL1A production by monocytes or monocyte-derived dendritic cells (DC) [4, 13]. T cell receptor (TCR) stimulation can upregulate TL1A on the surface of T cells, providing a source for autocrine TL1A-DR3 interactions in T cell activation [4].

Levels of TL1A have been reported to be elevated in serum and synovial fluid (SF) from RA patients [15, 16]. However, whether TL1A is elevated preferentially in RA versus other inflammatory diseases and whether the association of TL1A with RA simply reflects TL1A elevation in response to inflammation is not clear. TL1A can be induced by TNF stimulation of chondrocytes, synoviocytes, and endothelial cells [16, 17]. TL1A levels have been reported to fall in small cohorts of patients treated with TNF inhibitors [15, 18], but whether this is a direct consequence of TNF inhibition or secondary to clinical improvement after therapy is not known.

In this study, we investigated the triggers of TL1A production and examined the levels of TL1A in RA compared to other rheumatic and inflammatory joint diseases in multiple independent patient cohorts. We found that serum and synovial fluid TL1A levels are significantly more elevated in RA than in other rheumatic diseases and can be elevated in early disease and even those at high-risk for RA. In RA, TL1A declines after TNF blockade in both responders and non-responders, whereas TL1A only declines in responders to MTX treatment. In collagen-induced arthritis (CIA), we found that TL1A blockade is clinically effective and quantitatively blocks the development of bone erosions.

## Materials and methods

### Monocyte cell culture and stimulation

Elutriated monocytes from normal donors were obtained from the NIH transfusion medicine department under NIH IRB approved clinical protocols. Monocytes were cultured at 1 × 10^6^ cells/ml in RPMI medium with 10% FCS in a 37 °C, 5% CO_2_ incubator. At specified time points, culture supernatants were collected for measurement of TL1A and cells were harvested for qRT-PCR for measurement of TL1A mRNA. LPS (Ultrapure Salmonella Minnesota R595, List Biological Laboratories Inc., Campbell, CA) and other TLR ligands (Invivogen) were added at the indicated concentrations. Stimulation with immune complexes was performed as previously described [4].

### qRT-PCR

Quantitative RT-PCR was performed with the use of an ABI PRISM 7700 sequence-detection system with qScript One-Step qRT-PCR Kit, Low ROX (Quanta BioSciences, Inc.). Predesigned primer/probe sets were from Applied Biosystems, and the sequences designed to detect full-length TL1A are forward: 5′-CCCCGGAAAAGACTGTATGC-3′; reverse: 5′-GGTGAGTAAACTTGCTGTG-GTGAA-3′; and probe: 5′-TCGGGCCATAACAGAAGAGAGATCTGAGC-3′. Human IL-6 probe was from ThermoFisher (Hs00174131_m1). Each measurement was normalized to expression of β2-microglobulin (Themofisher Hs00187842_m1). The level of gene expression was calculated from 2^−ΔCt^. Gene expression levels were normalized to the level present in unstimulated cells.

### Patient cohorts

Multiple independent patient cohorts were included in this study. Two natural history cohorts of arthritis patients were used. One consisted of synovial fluid (SF) and plasma samples obtained simultaneously from 31 RA patients and 31 patients with other forms of arthritis at the Los Angeles County Hospital and University of Southern California Medical Center (IRB approved protocol HS-05-00270). Underlying causes of joint effusions were determined by medical history, physical examination, and SF analyses. A second, independent confirmation cohort (SNUH cohort) comprised 98 RA and 30 osteoarthritis (OA) patients receiving care at the Seoul National University Hospital between May 2010 and August 2012. All RA patients were over 18 years of age and satisfied 1987 American College of Rheumatology (ACR) classification of RA. OA patients were over 40 years of age and had a clinical diagnosis of OA for greater than 6 months. Patients with cancer, diabetes mellitus, coronary artery disease, and/or active infection were excluded. Study approval was given by the Seoul National University Institutional Review Board (C-1208-062-421). Concurrent SF and serum samples were available from 35 RA and 27 OA patients from this cohort. Clinical parameters such as joint counts, patient’s global health, erythrocyte sedimentation rate (ESR), high sensitivity C-reactive protein (hsCRP), RF, and anti-cyclic citrullinated peptide (CCP) antibody were acquired at the time of sampling. Demographic characteristics of this cohort are shown in Supplemental Table 1. Matched de-identified serum and SF samples were also obtained from discarded de-identified patients with osteoarthritis (OA), RA, psoriatic arthritis (PsA), gout, and pseudogout at the Brigham and Women’s Hospital Rheumatology clinic. Serum samples shown in Fig. 3c were from multiple sources: RA samples were provided by Dr. Raphaela Goldbach-Mansky under NIH protocol 00-AR-0222. Sjögren’s syndrome patient serum samples were obtained by Dr. Gabor Illei under NIH protocols 99-D-0070 and 84-D-0056. Ankylosing spondylitis (AS) patient serum samples were provided by Dr. Michael Ward under NIH protocol 03-AR-0131. Healthy control sera were obtained from the NIH blood bank. Sera from patients with systemic lupus erythematosus (SLE) were provided by Mary Crow and Kiriakos Kirou, Hospital for Special Surgery, New York, NY. Demographic data and SLE disease activity data (SELENA-SLEDAI) were recorded at the time of their clinical visit. Samples from patients with hepatitis C were obtained under NIH protocol 91-DK-0214.

Samples obtained from the SERA cohort [19] (Colorado Multiple Institutional Review Board approved protocol 01-675) included 198 first-degree relatives (FDRs) of patients with a confirmed diagnosis of RA who were themselves without inflammatory arthritis (IA) based on a 66/68 joint examination but who are at-risk for future RA based on family history (to be termed at-risk herein). SERA FDRs were randomly selected based on serum anti-cyclic citrullinated peptide (CCP) status as measured by CCP2 (Diastat, Axis-Shield, Diagnostics Ltd., Dundee, UK) and/or CCP3.1 (IgG/IgA, Inova Diagnostics Inc., San Diego, CA, USA) assays and included 78 anti-CCP-positive and 120 anti-CCP-negative FDRs. We also included 94 SERA subjects with anti-CCP-positive RA based on 1987 ACR and/or 2010 ACR/European League Against Rheumatism (EULAR) criteria. These RA subjects were divided into two sub-groups based on RA disease duration. We included 44 who were within 1 year of RA diagnosis (early RA) and 50 who had disease duration > 1 year (chronic RA). We also included healthy control subjects from the SERA cohort who were anti-CCP negative, without RA or inflammatory arthritis, and not a FDR with RA, who were recruited from the community through local advertisement. Demographic characteristics of these samples are listed in Supplemental Table 2.

Two cohorts of RA patients treated with TNF inhibitors were used. The first, shown in Fig. 4a, was drawn from the Autoimmune Biomarkers Collaborative Network (AbCoN) consortium [20] with 49 serum samples from TNF-naïve RA patients prior to and 14 weeks following initiation of anti-TNF therapy. Responders were classified by the EULAR criteria for change in DAS28 (ΔDAS28) as previously described [20]. Briefly, good responders had a ΔDAS28 ≥ 1.2 and DAS28 at 14 weeks ≤ 3.2; moderate responders are patients with a ΔDAS28 ≥ 1.2 and DAS28 at 14 weeks > 3.2, or a ΔDAS28 between 0.6 and 1.2 and DAS28 at 14 weeks ≤ 5.1; nonresponders did not fulfill either good or moderate responder criteria. The second group of samples (Fig. 4b) was from the GO-FURTHER golimumab study (NCT00973479, EudraCT 2008-006064-11) [21], with serum samples obtained at week 0 and 14 from 100 patients treated with golimumab (2 mg/kg IV on weeks 0 and 4, and then q8 weeks). Clinical responses were measured at week 14 by EULAR RA response criteria as in the AbCoN cohort.

Serum samples from two treatment trials conducted by the Rheumatology and Arthritis Investigational Network (RAIN) were used to study the effects of methotrexate (MTX) on TL1A levels. The first cohort was made up of patients in clinical trials testing the effects of the antibiotics minocycline and doxycycline in combination with MTX (University of Nebraska IRB #519-00-FB, NCT00579644 and IRB #344-98). Only those treated with MTX were included in this dataset. Both studies included patients with RF-positive RA with a duration of between 6 weeks and 1 year naïve to DMARD therapy and on oral corticosteroid doses of not greater than 7.5 mg/day prednisone equivalent. Subjects were treated with MTX (17.5 mg/week), and serum and clinical evaluations were performed after 6 months of therapy. The second cohort was taken from the Predictors study, an open-label, prospective study of patients with RA naïve to MTX or other DMARD therapy (University of Nebraska IRB #385-07-FB). Inclusion criteria included definite RA with a minimum of 4 swollen and 4 tender joints using 28 joint count. A stable dose of glucocorticoids of up to 10 mg/day was allowed. MTX monotherapy was given at 15 mg/week with escalation to 20 mg/week for patients not in remission at 8 weeks. Clinical response was measured at 4 months and response defined by EULAR criteria as above. For comparison of change in TL1A, the value of TL1A at baseline was subtracted from the endpoint value, with values below the limit of detection assigned to half of the lowest detectable concentration.

### Measurement of soluble TL1A and other cytokines in serum, plasma, and synovial fluid

**Fig. 1:**
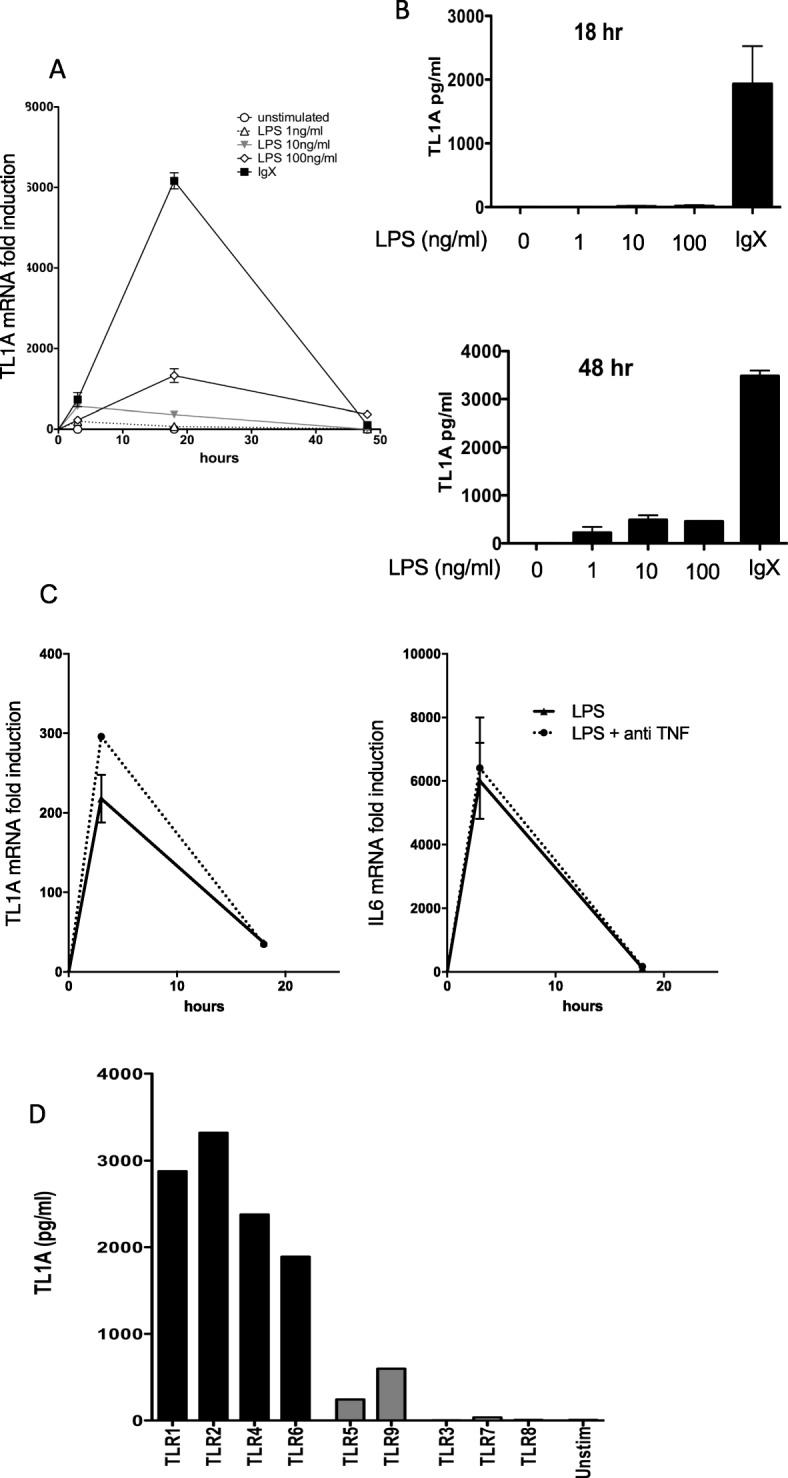
Induction of TL1A by immune stimuli in human monocytes. **a** Human peripheral blood monocytes (1 × 10^6^ cells/ml) were cultured with the indicated concentrations of LPS or with plate-bound immune complexes (IgX). Total RNA was isolated at the indicated time points, and TL1A gene expression was measured using qRT-PCR. TL1A mRNA levels were measured relative to human β2-microglobulin and normalized to that in resting monocytes and are shown as mean ± s.e.m. Data is representative of 5 independent experiments with different donors. **b** Monocyte culture supernatants were collected at 18 h and 48 h, and TL1A levels were measured using ELISA as described in the methods. Results in each panel are mean ± s.e.m. and are representative of a minimum of three experiments. Data is representative of results from 5 (LPS) and 3 (IgX) independent donors. **c** Peripheral blood monocytes were treated as in **a** with (100 ng/ml) LPS in the presence or absence of anti-TNF (10 μg/ml) and induction of TL1A and IL-6 measured by qRT-PCR. Data is representative of two independent experiments with different donors. **d** Monocytes at a higher initial concentration (2 × 10^6^ cells/ml) were stimulated with the indicated TLR agonist, and TL1A was measured in the supernatant after 48 h with a bead-based assay as described in the methods. Results are from one of two independent experiments with different donors

**Fig. 2:**
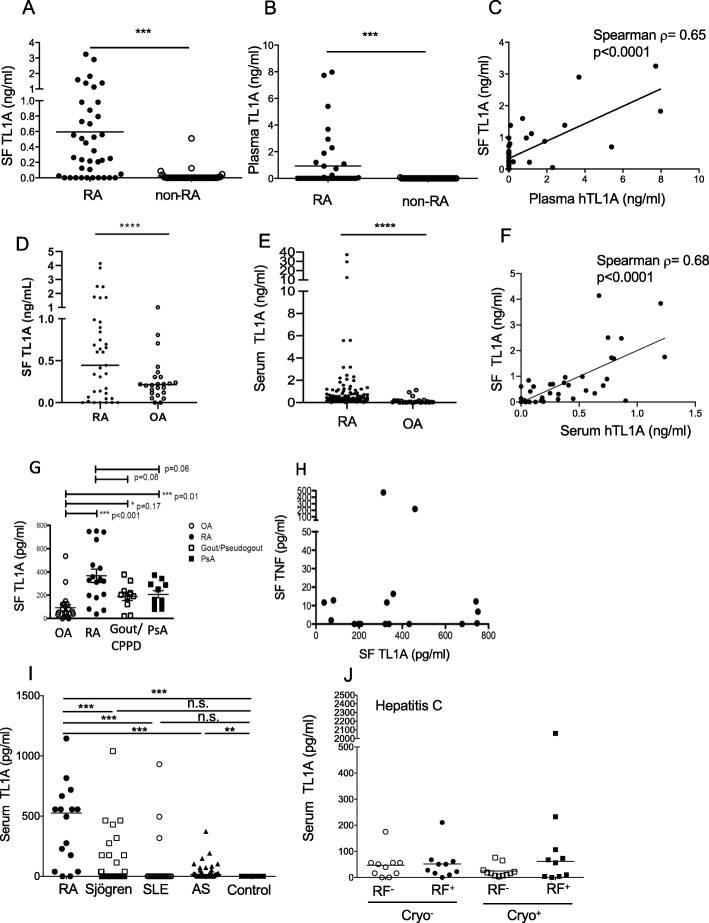
TL1A elevation in rheumatoid arthritis and other diseases. **a** Soluble TL1A was measured in SF from patients diagnosed with RA (*n* = 39) or other types of arthritis (non-RA, *n* = 37) including psoriatic arthritis, crystal-induced arthritis, reactive arthritis, non-inflammatory arthritis, nonspecific inflammatory arthritis, JIA, or infectious arthritis. Values are shown with a line representing the median value and significance of the difference between RA and non-RA samples *p* ≤ 0.0001 (Mann-Whitney test). **b** TL1A was measured in matching blood plasma samples as in **a**. *p* = 0.0002 (Mann-Whitney test). **c** TL1A levels in matched synovial fluid and plasma samples from RA patients (*n* = 39) were compared. Non-parametric correlation of SF vs. plasma TL1A is shown with the indicated Spearman’s *ρ* and *p* value. **d** Soluble TL1A was measured in synovial fluid (SF) from patients diagnosed with RA (*n* = 34) and osteoarthritis (OA) (*n* = 27). Values are shown with a line representing the median value and significance of the difference between RA and OA samples. *p* = 0.143 (Mann-Whitney test). **e** TL1A was measured in serum samples from patients diagnosed with RA (*n* = 98) and OA (*n* = 30) ****p ≤ 0.0001 (Mann-Whitney test). **f** TL1A levels in matched synovial fluid and serum samples from RA patients (*n* = 34) were compared. One outlier with very high TL1A in both serum (5.6 ng/ml) and SF (8.4 ng/ml) was removed from the analysis. Non-parametric correlation of SF vs. serum TL1A is shown with the indicated Spearman’s *ρ* and *p* value. For all panels. **g** TL1A was measured in SF from patients diagnosed with the indicated diseases using a bead-based immunoassay as described in the methods. RA *n* = 17; OA, *n* = 21; gout/CPPD (calcium pyrophosphate disease), *n* = 10; PsA (psoriatic arthritis), *n* = 11. Asterisks represent significances compared to OA (Mann-Whitney test) *p ≤ 0.05, ***p ≤ 0.001. **h** TL1A and TNF levels in SF from RA patients were measured by a bead-based assay as described in the methods. **i** Serum TL1A was measured as in **g** from patients diagnosed with the indicated diseases: RA, *n* = 20; AS ankylosing spondylitis, *n* = 40; Sjögren’s syndrome, *n* = 59; SLE systemic lupus erythematosus, *n* = 20; control, *n* = 10. Asterisks represent *p* value compared to control group **p ≤ 0.01, ***p ≤ 0.001 (Mann-Whitney test). **j** TL1A was measured in the serum of patients with hepatitis C with the indicated status for circulating cryoglobulins and/or rheumatoid factor

**Fig. 3:**
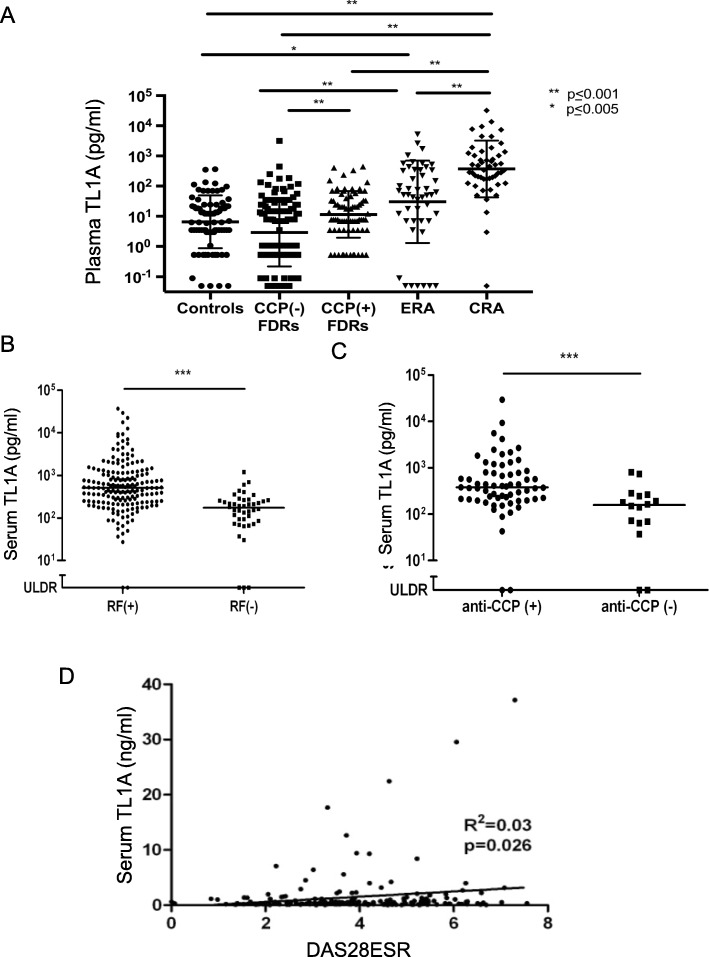
Relationship between circulating TL1A and disease duration, serological markers, and clinical activity in RA. **a** Plasma TL1A measured in the indicated groups of subjects from the SERA cohort. FDR: first-degree relatives grouped into those with or without anti-CCP antibodies. ERA, early RA (< 1 year); CRA, chronic RA (> 1 year). Significance of differences given by Mann-Whitney test with Bonferroni correction for multiple testing. Serum TL1A in RA patients from the SNUH cohort grouped by RF positivity (**b**) and anti-CCP positivity (**c**). Significance of the difference given by Mann-Whitney test. *p ≤ 0.05, **p ≤ 0.01,***p ≤ 0.001, ****p ≤ 0.0001. **d** Linear correlation of serum TL1A and DAS28 CRP in the SNUH RA cohort

**Fig. 4:**
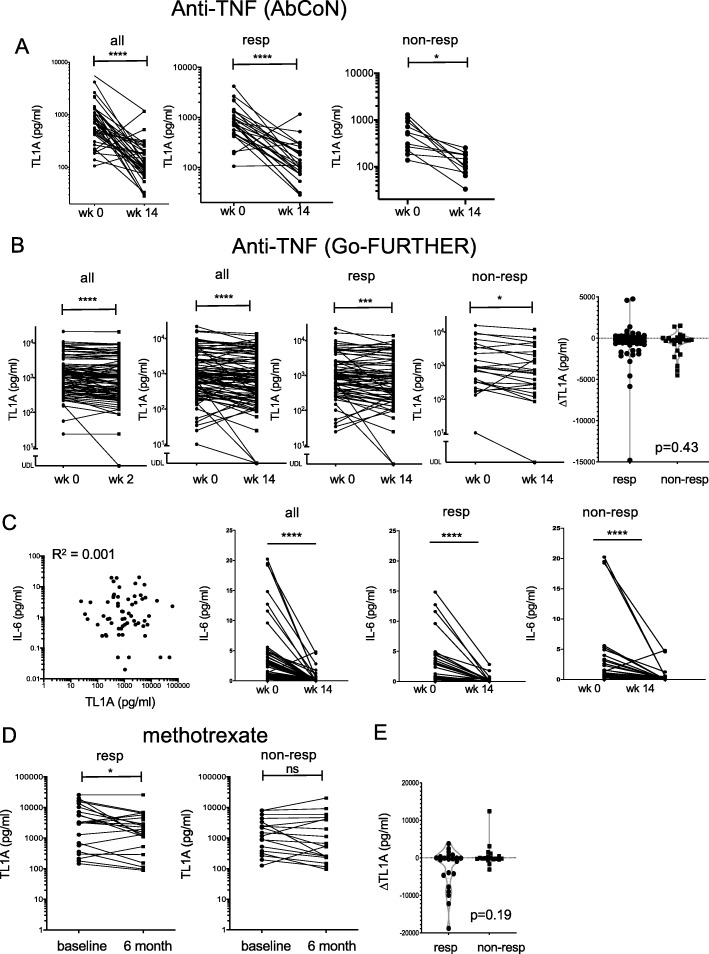
Effects of anti-TNF and MTX treatment on TL1A levels in rheumatoid arthritis. **a** Soluble TL1A was measured in serum samples from the AbCoN cohort [20] before or 14 weeks after treatment with TNF inhibitors. Subjects were classified by EULAR criteria into responders (good and moderate response, *n* = 34) and non-responders, *n* = 15. Significance for a change in TL1A was measured by Wilcoxon signed rank analysis. **b** Similar analysis was performed in a larger cohort (*n* = 100) of RA patients treated with golimumab, with serum TL1A levels shown at baseline compared to week 2 in all patients and week 14 in all patients, and grouped into responders (76) and non-responders (24) by EULAR criteria as in **a**. The right panel shows change in TL1A (14 weeks baseline) compared between responders and non-responders. The significance of the difference in ΔTL1A between responders and non-responders is given (Mann-Whitney non-parametric test). **c** Serum IL-6 measured in the same cohort of RA patients. Correlation with TL1A levels at baseline (left) and IL-6 levels at baseline and week 14 in all patients, and grouped into responders and non-responders by EULAR criteria as in **b** (right). **d** TL1A was measured in the serum before (baseline) or after 6 months of treatment with MTX monotherapy (17.5 mg/week) in 41 patients with early RA. Responsiveness was measured by EULAR criteria as described in the methods (23 good/moderate responders, 18 non-responders). **e** The change in TL1A (6 months baseline) was compared between responders and non-responders identified in **d**. The significance of the difference in ΔTL1A between responders and non-responders is given (Mann-Whitney non-parametric test)

A commercially available human TL1A ELISA kit (PeproTech, cat no. 900-K290) was used to measure TL1A levels in cell culture supernatants, sera, and synovial fluid in Fig. 1 and Fig. 2d–f. For measurement of TL1A in plasma and SF samples in Fig. 2a–c, an ELISA assay was developed using mouse anti-Human TL1A (clone 1A9) paired with biotinylated polyclonal anti-TL1A (PeproTech). For experiments shown in Fig. 2g–j, Fig. 3b-d, and Fig. 4a, a bead-based assay system was developed using anti-human TL1A antibody (clone 1A9) conjugated onto Bio-Plex COOH beads (Bio-Rad) and biotinylated polyclonal anti-human TL1A (Peprotech) for detection. For data shown in Figs. 3a and 4b, d, e and Figure S1 (additional file Figure S1.pdf), polyclonal anti-human TL1A conjugated to Bio-Plex beads was substituted for the 1A9 mAb, which allowed increased sensitivity of detection of TL1A in samples which had undergone multiple freeze thaws. Data was analyzed using the Prism (GraphPad Software, Inc.) software. For measurement of TNF, the Bio-Plex Pro Human Cytokine TNF-α set was used according to the manufacturer’s instructions (Bio-Rad, Cat no. 171-B5026M). For measurement of IL-6, the Bio-Plex Pro Human Cytokine IL-6 set was used according to the manufacturer’s instructions (Bio-Rad, Cat no.171BK29MR2).

### Induction of CIA and assessment of joint damage

Male DBA/1 J mice (8–10 weeks old) obtained from Jackson Laboratory (Bar Harbor, ME) were injected intradermally with 100 μg chicken type II collagen (CII) in complete Freund’s adjuvant (1:1, w/v) to the tail base at day 0 and boosted with an intra-dermal injection of 100 μg CII in incomplete Freund’s adjuvant (1:1, w/v) on day 21. Mice were injected i.p. with 20 mg/kg of hamster anti-mouse TL1A antibody (clone 5G4.6 [12]) or hamster immunoglobulin control every 7 days starting on day 21. Mice were euthanized at day 49. Animals were used under protocols approved by the NIAMS animal care and use committee. Development of arthritis was evaluated by macroscopic scoring of each paw on a 0–4 scale previously described [22]. Scoring was done by two separate investigators blinded to the treatment group, and the average score of each mouse was used. Anti-collagen antibody responses were measured by ELISA on plates coated with 50 μg/mL of chicken CII detected with 5 ng/mL goat anti-mouse HRP (Pierce, cat. no. 1858413). Micro-CT on formalin-fixed hind-limbs was performed with a SkyScan 1172 Micro X-ray CT scanner (MicroPhotonics, Inc. Allentown PA, USA, SkyScan, Kontich, Belgium) with the X-ray source (focal spot size, 4 μm, energy range 20–100 kV) biased at 44 kV/22 μA and with a 0.5-mm aluminum filter to reduce beam hardening. Images were acquired with a voxel size of 12.17 μM^3^, a camera to source distance of 220 mm, and an object to source distance of 116 mm. Four hundred fifty projections were acquired with an angular resolution of 0.4° through 180° rotation. Eight frames were averaged for each projection radiograph with an exposure time of 295 ms per frame. Tomographic images were reconstructed using vendor-supplied software based on the Feldkamp cone beam algorithm. Reconstructed images were then made into three-dimensional image using CTAn (v.1.10), then visualized using CTVol (v.2.1) (SkyScan). Three-dimensional images were scored on de-identified 3-D reconstructions by two separate investigators based on the scoring system described in Supplemental Figure 2.

## Results

### Induction of TL1A expression in human monocytes

In RA, FcR crosslinking by immune complexes and stimulation of TLRs by endogenous ligands may sustain inflammation by stimulating monocytes and other innate immune cells in the joint [23–25]. We tested the ability of immune complexes and TLR ligands to induce expression of TL1A by human monocytes. Both FcR crosslinking and lipopolysaccharide (LPS) in higher concentrations induced TL1A at the mRNA level, with levels peaking at 18 h and then rapidly declining (Fig. 1a). The peak of TL1A induction was ~ 6-fold higher following FcR crosslinking than following optimal concentrations of LPS (Fig. 1a). At the protein level, FcR crosslinking induced higher levels of TL1A that were detectable at 18 h, whereas LPS-induced TL1A was only detectable at 48 h, with TL1A production approximately 10-fold lower with optimal doses of LPS than with FcR crosslinking (Fig. 1b). Induction TL1A by LPS was also evident at the mRNA level, with a similar time course as IL-6 (Fig. 1c). Induction of both TL1A or IL-6 was independent of TNF secretion, as addition of anti-TNF to these cultures did not reduce induction of mRNA (Fig. 1c). TLRs can be divided into subclasses depending on whether they activate signaling pathways at the cell surface (TLR 1, 2, 4, 5, and 6) or endosomally (TLR 3, 7, 8, and 9). To determine which of these signaling pathways are important in induction of TL1A expression, we stimulated human monocytes with a panel of TLR ligands specific for each receptor. Stimulation through TLR 1, 2, 4, and 6 were the most efficient at inducing TL1A; TLR 5 and 9 were intermediate; and stimulation through TLR 3, 7, and 8 did not induce detectable TL1A production (Fig. 1d). These data identify FcR and TLRs which are present at the cell surface as the most potent inducers of TL1A secretion by human monocytes.

### TL1A is elevated in rheumatoid arthritis serum and synovial fluid

To determine the relationship between synovial fluid (SF) and blood TL1A levels in RA, and the specificity of TL1A elevation for RA, we measured TL1A in matched samples of SF and plasma from a cohort of patients with RA (39 samples from 31 patients) or other types of arthritis (37 samples from 31 patients), including psoriatic arthritis, crystal-induced arthritis, reactive arthritis, OA, nonspecific inflammatory arthritis, juvenile inflammatory arthritis (JIA), or infectious arthritis. The majority (27 of 39) of SF samples from RA patients had TL1A levels above 0.1 ng/mL, with a mean of 0.59 ng/mL and up to 3.25 ng/mL detected. By contrast, only 2 of 37 non-RA samples (one each from a patient with reactive arthritis and a patient with psoriatic arthritis) contained more than 0.1 ng/mL TL1A (Fig. 2a). In plasma, a smaller but still significant percentage of RA patients had elevated TL1A levels, while no samples from patients with other arthritides had detectable TL1A levels (Fig. 2b). Of note, there was a modest correlation between plasma and SF TL1A levels in 39 matched RA plasma and SF samples (Fig. 2c, Spearman’s *ρ* = 0.65, *p* < 0.0001). Taken together, these results show that TL1A levels are preferentially elevated in RA compared to other forms of inflammatory arthritis. To confirm these findings in a second, independent cohort of patients, we measured serum TL1A levels in a group of 98 RA and 30 OA patients, with matched SF samples available from some patients (Fig. 2d–f). TL1A in SF from these RA patients trended towards being elevated over OA, and serum TL1A was significantly elevated in RA vs. OA patients. TL1A in RA serum also correlated with TL1A levels in SF (Spearman’s *ρ* = 0.68, *p* < 0.0001). We also examined SF TL1A in a third cohort of patients with RA and other defined rheumatic diseases, finding that TL1A is most elevated in RA and PsA compared to OA, but not in crystal arthropathies (Fig. 2g). Levels of soluble TNF and TL1A did not correlate in the SF samples from patients with RA (Fig. 2h).

To further explore the elevation of TL1A levels in RA compared with other autoimmune disorders, we measured TL1A levels in sera from patients with Sjögren’s syndrome (SS) and systemic lupus erythematosus (SLE) and compared these to serum from an independent cohort of RA patients and serum from healthy volunteers. TL1A levels were significantly elevated in patients with RA (median 525.7 pg/ml) compared to healthy controls, none of whom had detectable serum TL1A (Fig. 2i). Serum TL1A levels were non-significantly elevated in Sjögren’s syndrome compared to controls, and TL1A was undetectable in the serum of all but three SLE patients. Interestingly, each of these 3 SLE patients had high SLEDAI scores (range, 8–12), and two had arthritic flares at the time of sample collection. There was no correlation between elevated TL1A levels and autoantibody titers or evidence for circulating immune complexes such as depressed complement levels or nephritis among those with SLE. As an additional control, we measured TL1A in the serum of patients with ankylosing spondylitis (AS), which is not associated with autoantibodies or circulating immune complexes. Although the median TL1A level in AS serum (14.5 pg/ml) was significantly higher than that in healthy controls (undetectable), only a minority of AS serum contained TL1A at levels higher than 100 pg/ml.

Elevated levels of TL1A seen in RA may also occur in other diseases associated with circulating immune complexes and RF expression. To determine if RF or immune complexes produced in other disease states could induce TL1A, we measured TL1A in serum from patients with hepatitis C with or without RF or circulating cryoglobulins (Fig. 2j). TL1A levels were higher than 100 pg/ml in more patients with both cryoglobulins and RF than the other groups, but this increase was not statistically significant, and the median TL1A level in RF-positive hepatitis C patients (27.9 pg/ml) was much lower than in RA. No correlation was seen between serum TL1A and hepatitis C viral load, serum liver enzyme levels, liver biopsy, or tests of hepatic synthetic function (albumin and prothrombin time, data not shown). Taken together, these results indicate that serum levels of TL1A are more elevated in RA than in other rheumatic diseases and other diseases marked by circulating immune complexes.

### TL1A levels are increased in high-risk family members and in early RA and correlate with seropositivity

Development of anti-CCP and RF pre-date the clinical onset of seropositive RA, and in combination with the presence of pro-inflammatory cytokines in the serum, predict a higher risk for the future development of classifiable RA [26, 27]. To determine whether elevated TL1A is also present in the at-risk population and in early vs. established RA, we measured serum TL1A in a cohort of 198 first-degree relatives (FDR) of RA patients without inflammatory arthritis and compared these levels to 44 patients with early RA (ERA; < 1 year in duration), 50 patients with chronic RA (CRA; > 1 year in duration), and 80 healthy non-FDR controls. Anti-CCP antibody-positive FDRs had significantly higher TL1A than anti-CCP-negative FDRs and healthy controls (Fig. 3a). In patients with early RA, serum TL1A was also higher than in controls, and TL1A levels were significantly higher in patients with chronic RA than in those with early RA (Fig. 3a). These data show that increased serum TL1A can be an early event during the development of RA and the levels rise with increasing duration of disease. In an independent cohort of RA patients, serum TL1A levels were also significantly higher in those with RF or anti-CCP (Fig. 3b, c). However, clinical measures of disease activity such as the DAS28 were only weakly associated with TL1A levels (Fig. 3d).

### Circulating TL1A in RA depends on TNF

TNF can induce TL1A both ex vivo in fibroblast-like synoviocytes, and in mouse models of inflammatory bowel disease [16, 28], raising the possibility that TL1A expression may be under the control of TNF in RA. To test whether serum TL1A levels depend on TNF in an in vivo clinical setting, we examined serum TL1A levels before and after initiation of anti-TNF therapy in samples from 49 RA patients in the Autoimmune Bio-markers Collaborative Network (AbCoN) cohort, which was designed to test predictors of responsiveness to TNF blockade [20, 29]. As in the other cohorts of RA patients, TL1A was significantly elevated (mean, 747.5 ± 119 pg/ml), but was not significantly correlated with ESR, CRP, or joint counts at baseline. Fourteen weeks of TNF blockade dramatically lowered serum TL1A levels (156.1 ± 26 pg/ml, Fig. 4a), with TL1A declining by an average of 27% in an individual patient after TNF blockade and TL1A falling in all but two of the patients after initiation of TNF inhibitor therapy. To confirm these results, we studied a larger cohort of 100 RA patients treated with the fully human monoclonal anti-TNF antibody golimumab in a 14-week clinical trial (Fig. 4b). The magnitude of the decline in TL1A in response to TNF blockade was smaller in this cohort (mean TL1A 2357 pg/ml at week 0, 1799 pg/ml at week 14, 19% average decline), but as in the AbCoN cohort, TL1A declined significantly at 14 weeks in both responders and non-responders. The decline in TL1A was already seen after 2 weeks (Fig. 4b, left panel). Baseline TL1A levels, CRP, or DAS28 were not predictive of a response to TNF blockade in either cohort. The magnitude of the change in TL1A was not significantly different between responders and non-responders (Fig. 4b, right panel). We also measured serum IL-6 in these samples, finding that TL1A was not correlated with IL-6 at baseline (Fig. 4c, left panel). Although absolute levels of IL-6 were lower than TL1A, IL-6 also fell in response to TNF blockade irrespective of clinical response status (Fig. 4c, right panels).

The fall in serum TL1A early after initiation of TNF blockade and irrespective of treatment outcome suggested that serum TL1A may be under the direct control of TNF. If this were the case, then treatment with another agent not acting through TNF might only lead to a decrease in TL1A level in responders. We tested this in two independent cohorts of patients with early RA treated with MTX. In both cohorts, the reduction in TL1A in an individual subject was only significant in MTX responders, but the magnitude of the change in TL1A was not significantly different (Fig. 4d, e, S1, additional file Figure S1.pdf). These data suggest that the decline in TL1A after MTX treatment in a responder may be due to the reduction in joint inflammation but cannot be distinguished from indirect effects of MTX therapy on TL1A.

### Blocking TL1A-DR3 interactions improves clinical outcome and bony erosions in collagen-induced arthritis

**Fig. 5:**
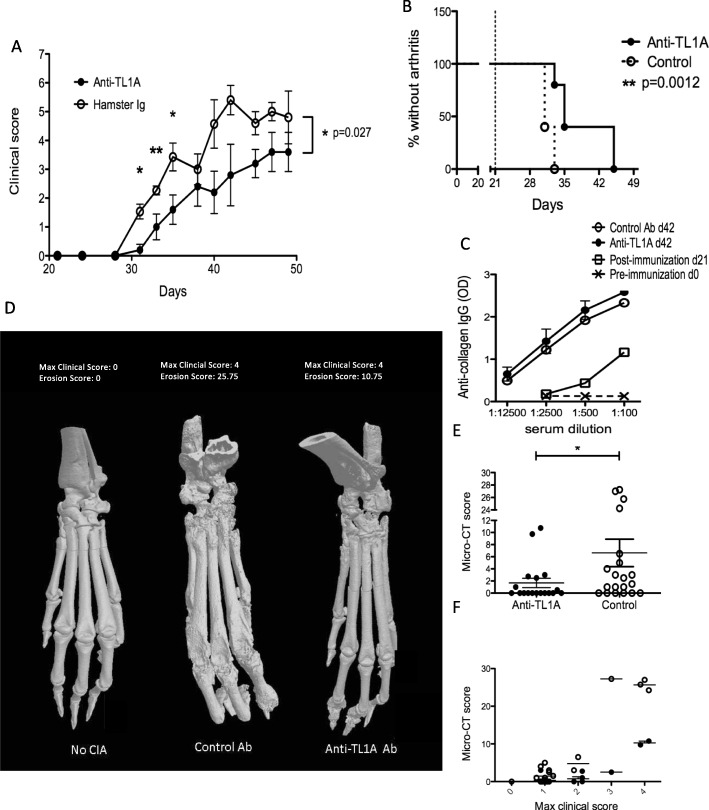
Blocking TL1A-DR3 interactions prevents disease and bone erosions in collagen-induced arthritis. **a** CIA was induced in DBA/1 mice as described in the methods. Weekly intra-peritoneal injections of 20 mg/kg of either anti-TL1A (treatment, *n* = 5) or hamster immunoglobulin Ig control (*n* = 7) were begun at day 21 after initial immunization with collagen. A representative experiment of three independent experiments is shown. Clinical scores on each day were compared using an unpaired *t* test, and *p* values for significance are shown above each time-point represented by asterisks (*p < 0.05, **p < 0.01) above each day. Two-way ANOVA was also performed to compare the trend of the two graphs, with *p* value shown to the right. **b** Survival analysis of the percentage of mice without arthritis on each day is compared between the anti-TL1A treated group and control group. Arthritis was defined by a combined clinical score of two or more. **c** Sera from mice from each group induced to develop CIA as in **a** were collected at indicated time points and anti-chicken collagen IgG levels were measured by ELISA. **d** 3-D reconstructions of micro-CT examination of hind paws from mice induced to develop CIA with and without TL1A blockade. Examples are shown from each treatment group, with the maximal clinical scores and the erosion score obtained for that paw by two separate observers blinded to treatment groups. **e** Composite of CT erosion scores obtained from the anti-TL1A treated group (*n* = 18) and control-antibody treated group (*n* = 20), significance is from an unpaired *t* test with Welch’s correlation (*p ≤ 0.05). Analysis of individual regions resulted in *p* values of 0.078 at ankle/tarsus, 0.042 at metatarsophalangeal (MTP) joints, and 0.015 at toes. **f** Comparison of the CT scores of the paws from the two groups based on the maximum clinical scores. Anti-TL1A treatment significantly reduced erosions independent of the clinical score. *p* < 0.0001 by 2-way ANOVA

The reduction in serum TL1A levels after anti-TNF treatment of RA suggests that TL1A may be a biomarker for TNF activity in RA but does not indicate whether TL1A contributes to RA pathogenesis. To investigate this question, we turned to the CIA mouse model of RA. Mice treated with a blocking antibody against mouse TL1A during the induction of arthritis had a significant reduction in total joint score for the ensuing 28 days, especially at earlier time points. The onset of measurable clinical signs of arthritis (joint score ≥ 2) was significantly delayed by anti-TL1A (Fig. 5a, b). Interestingly, the decrease in the severity of arthritis in mice treated with anti-TL1A was not associated with decreases in titers of anti- collagen antibodies (Fig. 5c). Taken together, these data show that blocking TL1A-DR3 interactions potently reduces the clinical inflammatory signs of CIA without affecting the systemic immune response to the collagen immunogen.

To assess whether anti-TL1A treatment also prevented bone erosions in CIA, we used micro-CT scanning to quantitate bony changes in the hind limbs. As shown in the examples in Fig. 5d, anti-TL1A treatment dramatically reduced erosions in the hind paws of mice induced to develop CIA. Using a scoring system developed to take into account erosions and deformities in each joint in the hind paws (Figure S2, additional file Figure S2.pdf), we found a significant reduction in average and maximum erosion scores in anti-TL1A-treated mice (Fig. 5e). The reduction was especially pronounced in the MTP joints (*p* = 0.042) and toes (*p* = 0.015). Strikingly, erosions in anti-TL1A treated mice were significantly reduced in paws with similar maximum clinical scores (Fig. 5f, 2-way ANOVA *p* < 0.0001 for treatment effect independent of maximum clinical score, *p* < 0.0001 for treatment and *p* < 0.0001 for maximum clinical score). This indicates that anti-TL1A antibody treatment not only diminished the erosions by inhibiting clinical arthritis but also provided protection against erosions independent of inflammation as measured by the clinical joint score.

## Discussion

Although the expression of multiple pro-inflammatory cytokines is elevated in RA, only blockade of TNF or IL-6 ameliorates disease symptoms and progression of erosions. The data presented here strengthens the evidence that the TNF-family cytokine TL1A is pathogenic in animal models of RA and clarifies the relationship between TL1A and TNF in RA. The strong induction of TL1A by FcR crosslinking is consistent with previous observations suggesting that TL1A can be induced by RF-containing immune complexes [14]. Our findings that TL1A can be induced by TLR 1, 2, 4, and 6 are consistent with previous results in murine DC where LPS induced TL1A [4], but in contrast with those of Prehn et al. [13], who found that only FcR, not TLR ligands, could induce TL1A secretion by human monocytes.

The increased circulating serum TL1A in RA compared to other rheumatic diseases suggests that TL1A is not simply being produced in response to inflammation. The correlation between serum and synovial fluid TL1A is consistent with other studies [30] and supports a synovial origin for circulating TL1A. Our findings confirm previous reports of elevated blood and SF TL1A levels in smaller cohorts of RA patients [15, 16, 30] and demonstrate the increased magnitude of TL1A elevation in RA vs. other inflammatory diseases, though serum TL1A is still elevated in some other diseases compared to healthy volunteers. We found a low but significant elevation of TL1A in AS sera compared to healthy controls. Elevated serum TL1A has been reported in an AS [31], and *TNFSF15* polymorphisms have been suggested to be linked to the risk for SpA and AS [32, 33]. Serum TL1A has previously been reported to be elevated in SLE and to a lesser extent, in systemic sclerosis [34, 35], but it is difficult to compare the degree of elevation in serum TL1A across studies. The lack of consistent elevation of TL1A in patients with hepatitis C even in the presence of circulating immune complexes and/or RF suggests that there may be additional stimuli for TL1A production in RA, such as activation of Toll-like receptors by endogenous ligands in the joint [36]. RF levels in hepatitis may not be sufficient to trigger TL1A production, or release of TL1A into the circulation may require the metalloprotease-rich environment of the inflamed joint. The increased levels of TL1A in CCP(+) or RF(+) patients that we found is consistent with previous results in established RA [15, 30]. The elevation of TL1A in anti-CCP(+) at-risk relatives of RA patients shows for the first time that TL1A elevation can precede the diagnosis of RA and also raises the possibility that TL1A levels, either in cross sectional or longitudinal studies, may be a predictive biomarker of progression to RA, although a larger study would be needed to confirm this.

Our findings suggest a close relationship between TL1A and TNF in inflammatory arthritis. As in prior smaller studies [15, 18], TL1A levels fell after TNF blockade in RA. We show here for the first time that TL1A levels fall regardless of the clinical response to TNF blockade in RA in two independent RA treatment cohorts. This suggests that TL1A is directly or indirectly under the control of TNF in RA. These findings were confirmed in two independent cohorts for each treatment; but it should be noted that these studies were carried out independently and with different inclusion criteria, so other confounding factors may influence these results. The dependence of TL1A on TNF is not unique to TL1A, as we showed that IL-6 also falls after TNF blockade. This data is in line with animal experiments showing that TNF can induce TL1A expression in endothelial cells, chondrocytes, and synovial fibroblasts [16, 17] Although pre-treatment levels of TL1A did not predict responsiveness to anti-TNF, a study of other cytokines in the AbCoN cohort of RA patients before anti-TNF treatment also did not identify any single cytokine that could predict anti-TNF responsiveness [29], and even measurement of TNF itself has not consistently shown to be associated with responsiveness to TNF blockade in RA, possibly because of the very low levels of TNF (< 10 pg/ml) found in the serum of most RA patients [37–40]. Further studies would be needed to determine whether TL1A in combination with other cytokines or biomarkers in combination may predict responsiveness to TNF inhibition.

In the CIA mouse model of RA, blocking TL1A-DR3 interactions using an anti-TL1A mAb reduced clinical severity of arthritis and provided robust protection of joints from bony erosions. These results are consistent with prior results in this model with other blocking anti-TL1A antibodies [11] and in DR3-deficient mice crossed to the CIA-sensitive DBA/1 background [41]. Interestingly, TL1A blockade in CIA did not suppress the secondary antibody response to collagen, supporting findings in other T cell-dependent models of autoimmune disease that TL1A-DR3 interactions support T cell responses at the site of inflammation and are dispensable for systemic immune responses to immunogens [42]. The reduction in clinical arthritis score is within in the range of that observed for TNF and IL-1 blockade [43], but the comparison should be interpreted with caution as the severity of the CIA model is variable between experiments and laboratory environments. Our observation that anti-TL1A reduced erosions independent of the joint clinical score is novel and reminiscent of results seen with anti-TNF agents in RA, where progression of erosions was inhibited independently of clinical improvement [1, 44]. However, efficacy of cytokine blockade in the CIA model is not predictive of that in RA [43]. Longer-term studies would be needed to address whether the fall in TL1A associated with TNF therapy in RA predicts delay in progression of erosions, or development of atherosclerosis, which has also been associated with higher serum levels of TL1A [45].

## Conclusions

These results show that TL1A is elevated in the serum of rheumatoid arthritis and becomes elevated even in at-risk FDR with anti-CCP antibodies. The decline in TL1A after TNF blockade in RA irrespective of clinical response suggests that circulating TL1A levels may be a biomarker for TNF activity in RA. The therapeutic effects of TL1A blockade on clinical score and bone erosions in CIA combined with previous data on efficacy of TL1A blockade or TL1A/DR3 deficiency in animal models of asthma, multiple sclerosis, and inflammatory bowel disease suggest that TL1A blockade should be further explored as a therapeutic strategy in these diseases.

## Supplementary information

**Additional file 1: Figure S1.** Analysis of effect of methotrexate on TL1A levels from a second independent patient cohort. TL1A was measured in serum from an independent cohort of patients with RA treated with MTX for 4 months (66 good/moderate EULAR responders, 18 non-responders).

**Additional file 2: Figure S2.** Micro-CT Scoring system for erosions and joint damage (MCTS). A scoring system was devised for micro-CT measurements of hind paw joint scores incorporating a score for the ankle/tarsus, MTP, and distal joints with a total score of 0–30 points.

**Additional file 3: Table S1.** Demographic characteristics of SNUH cohort (data shown in Fig. 2 D-F and Fig. 3 B-D). Sr, serum; SF, synovial fluid; ESR, erythrocyte sedimentation rate; hsCRP, high sensitive C-reactive protein; RF, rheumatoid factor. **Table S2.** Demographic characteristics of patient subgroups from the SERA cohort.

## Data Availability

Material described in this study will be made available to requesting parties.

## Abbreviations

AS: Ankylosing spondylitis
CCP: Cyclic citrullinated peptide
CIA: Collagen-induced arthritis
DC: Dendritic cell
ESR: Erythrocyte sedimentation rate
FcR: Fc receptor
FDR: First-degree relative
hsCRP: High sensitivity C-reactive protein
LPS: Lipopolysaccharide
MTX: Methotrexate
OA: Osteoarthritis
PsA: Psoriatic arthritis
RA: Rheumatoid arthritis
RF: Rheumatoid factor
SF: Synovial fluid
TCR: T cell receptor
TL1A: TNF-like protein 1A
TNF: Tumor necrosis factor
