# Supplementary material for: Circulating TNF-like protein 1A (TL1A) is elevated early in rheumatoid arthritis and depends on TNF
Source: Arthritis Res Ther. 2020 May 7;22:106. doi: 10.1186/s13075-020-02198-9 (PMC7204024; doi:10.1186/s13075-020-02198-9)
Supplement: Supplementary file 1 — Additional file 1: Figure S1. Analysis of effect of methotrexate on TL1A levels from a second independent patient cohort. TL1A was measured in serum from an independent cohort of patients with RA treated with MTX for 4 months (66 good/moderate EULAR responders, 18 non-responders). [file 13075_2020_2198_MOESM1_ESM.pdf]

Figure S1: Analysis of effect of methotrexate on TL1A levels from a second independent patient cohort

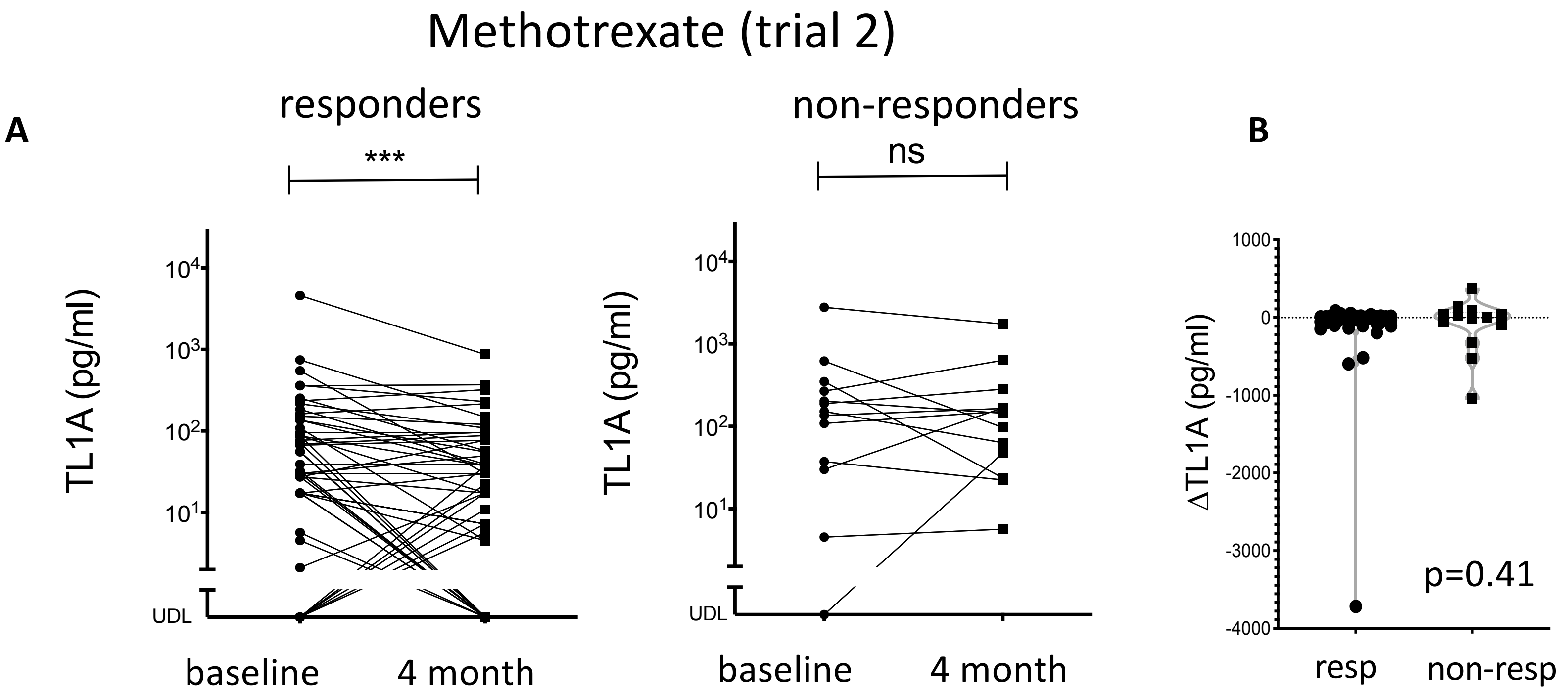

A. TL1A was measured in serum from an independent cohort of patients with RA treated with MTX for 4 months (66 good/moderate EULAR responders, 18 non-responders). (Significance for a change in TL1A level from baseline was measured by Wilcoxon signed rank analysis * $p<0.05$, *** $p<0.001$, **** $p<0.0001$ for all panels. B. The change in TL1A (4 months-baseline) was compared between responders and non-responders identified in A. The significance of the difference in ΔTL1A between responders and non-responders is given (mann-whitney test)
