# Supplementary material for: Circulating TNF-like protein 1A (TL1A) is elevated early in rheumatoid arthritis and depends on TNF
Source: Arthritis Res Ther. 2020 May 7;22:106. doi: 10.1186/s13075-020-02198-9 (PMC7204024; doi:10.1186/s13075-020-02198-9)
Supplement: Supplementary file 2 — Additional file 2: Figure S2. Micro-CT Scoring system for erosions and joint damage (MCTS). A scoring system was devised for micro-CT measurements of hind paw joint scores incorporating a score for the ankle/tarsus, MTP, and distal joints with a total score of 0–30 points. [file 13075_2020_2198_MOESM2_ESM.pdf]

Figure S2: Micro-CT Scoring system for erosions and joint damage (MCTS) maximum score: 30 per paw

- Ankle/Tarsus: 0 – 2 in 0.5 increments, then x5 → 10 max
  - Normal: 0
  - Mild: 0.5-1
  - Moderate: 1-1.5
  - Severe: 2
- MTP: 0, 1 or 2 per joint, 5 joints → 10 max
  - Normal (0)
  - Mild-Moderate (1): periosteal changes, or mild erosions, but joint space maintained
  - Severe (2): severe erosions, especially on both sides of the joint, grossly deformed
- PIP/Digits: 0, 1 per digit, 5 digit → 5 max
  - Normal (0)
  - Affected (1): periosteal changes or erosions
- Deformity → 0 or 5
  - Normal (0): overall structure mostly maintained
  - Deformed (5): ankylosis and gross deformity

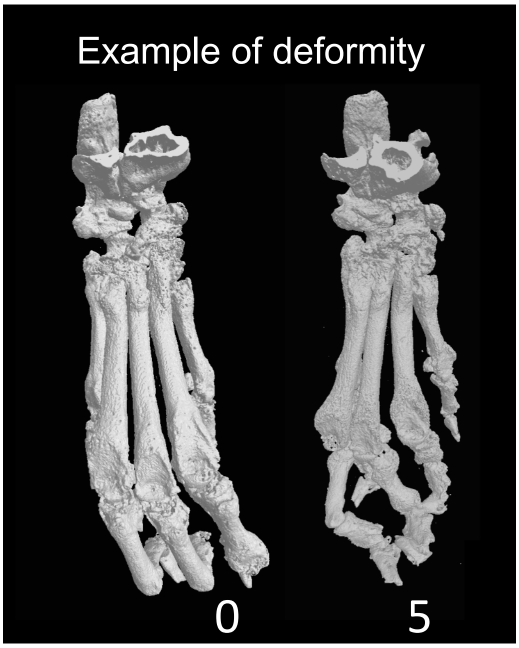

Examples of sub-scores

	Score 0	Score 1	Score 2
Ankle/Tarsus	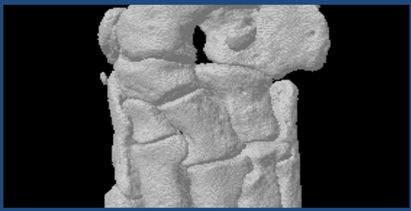	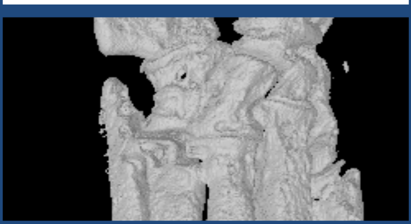	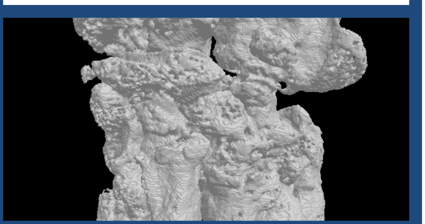
MTP	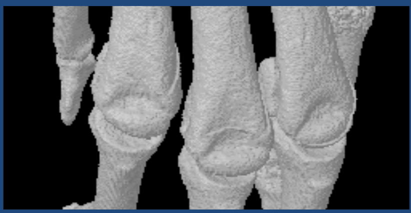	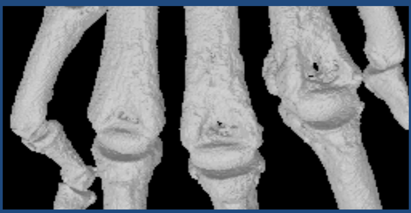	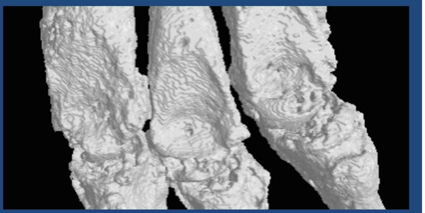
PIP/Digits	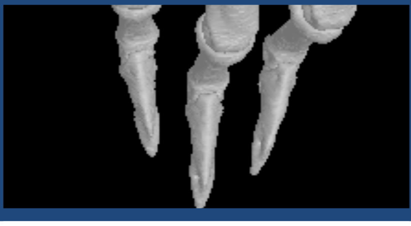	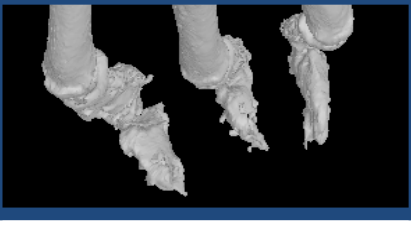	
