# Supplementary material for: Circulating TNF-like protein 1A (TL1A) is elevated early in rheumatoid arthritis and depends on TNF
Source: Arthritis Res Ther. 2020 May 7;22:106. doi: 10.1186/s13075-020-02198-9 (PMC7204024; doi:10.1186/s13075-020-02198-9)
Supplement: Supplementary file 3 — Additional file 3: Table S1. Demographic characteristics of SNUH cohort (data shown in Fig. 2 D-F and Fig. 3 B-D). Sr, serum; SF, synovial fluid; ESR, erythrocyte sedimentation rate; hsCRP, high sensitive C-reactive protein; RF, rheumatoid factor. Table S2. Demographic characteristics of patient subgroups from the SERA cohort. [file 13075_2020_2198_MOESM3_ESM.docx]

**Supplemental Table 1: Demographic characteristics of SNUH cohort (data shown in Figure 2 D-F and Figure 3 B-D)**

|  | RA (n=98) | Sr-SF matched RA (n=34) | OA (n=27) |
| --- | --- | --- | --- |
| Age (years) | 64.2 ± 6.5 | 62.5 ± 9.0 | 64.2 ± 10.2 |
| Female (%) | 91.2 | 94.4 | 92.6 |
| Disease duration (years) | 10.4 ± 8.7 | 11.5 ± 7.6 |  |
| ESR (mm/hr) | 37.7 ± 32.4 | 42. 3 ± 27.0 |  |
| hsCRP (mg/dL) | 1.7 ± 2.8 | 3.2 ± 2.9 |  |
| RF positivity (%) | 80.5 | 82.4 |  |
| Anti-CCP antibody positivity (%) | 86.1 | 85.0 |  |
| Radiographic change (%) | 74.0 | 82.8 |  |

Sr, serum; SF, synovial fluid; ESR, erythrocyte sedimentation rate; hsCRP, high sensitive C-reactive protein; RF, rheumatoid factor

**Supplemental Table 2: Demographic characteristics of patient subgroups from the SERA cohort**

| 1. **Overall demographics** | | | | |
| --- | --- | --- | --- | --- |
|  | Controls  n=80 | FDRs  n=198 | RA  n=94 | p-value |
| Age, mean ± SD | 37±13 | 53±17 | 56±14 | <0.01 |
| Female, (%) | 75.0 | 78.3 | 73.4 | 0.63 |
| Non-Hispanic white, (%) | 72.5 | 75.3 | 71.3 | 0.74 |
| Ever-smoker, (%) | 20.0 | 35.9 | 50.0 | <0.01 |
| Current-smoker, (%) | 5.0 | 6.6 | 16.0 | 0.01 |
| Shared epitope (≥1 allele), (%) | 36.8 | 53.5 | 72.2 | <0.01 |

FDR, first-degree relatives; RA, rheumatoid arthritis

| **B. RA first-degree relative subject characteristics by anti-CCP status** | | | |
| --- | --- | --- | --- |
|  | FDR  Anti-CCP(-)  n=78 | FDR  Anti-CCP(+)  n=120 | p-value |
| Age, mean ± SD | 55±17 | 52±17 | 0.27 |
| Female, (%) | 78.2 | 78.3 | 0.98 |
| Non-Hispanic white, (%) | 75.6 | 75.0 | 0.32 |
| Ever-smoker, (%) | 38.5 | 34.2 | 0.54 |
| Current-smoker, (%) | 6.4 | 6.7 | 1.0 |
| Shared epitope (≥1 allele), (%) | 55.1 | 52.5 | 0.72 |

RA, rheumatoid arthritis; FDR, first-degree relatives; Anti-CCP, anti-cyclic citrullinated peptide antibody

| **C. RA subject characteristics by disease duration** | | | |
| --- | --- | --- | --- |
|  | Early RA  n=44 | Chronic RA  n=50 | p-value |
| Age, mean ± SD | 52±14 | 59±13 | 0.01 |
| Female, (%) | 70.5 | 76.0 | 0.54 |
| Non-Hispanic white, (%) | 65.9 | 76.0 | 0.28 |
| Ever-smoker, (%) | 45.5 | 54.0 | 0.41 |
| Current-smoker, (%) | 18.6 | 14.0 | 0.55 |
| Shared epitope (≥1 allele), (%) | 66.7 | 77.1 | 0.27 |

RA, rheumatoid arthritis
